# A first insight into the involvement of phytohormones pathways in coffee resistance and susceptibility to *Colletotrichum kahawae*

**DOI:** 10.1371/journal.pone.0178159

**Published:** 2017-05-19

**Authors:** Inês Diniz, Andreia Figueiredo, Andreia Loureiro, Dora Batista, Helena Azinheira, Vítor Várzea, Ana Paula Pereira, Elijah Gichuru, Pilar Moncada, Leonor Guerra-Guimarães, Helena Oliveira, Maria do Céu Silva

**Affiliations:** 1 Centro de Investigação das Ferrugens do Cafeeiro (CIFC), Instituto Superior de Agronomia (ISA), Universidade de Lisboa, Oeiras, Portugal; 2 Linking Landscape, Environment, Agricultural and Food (LEAF), Instituto Superior de Agronomia (ISA), Universidade de Lisboa, Lisboa, Portugal; 3 BioISI-Biosystems and Integrative Sciences Institute, Faculdade de Ciências, Universidade de Lisboa, Lisboa, Portugal; 4 Computational Biology and Population Genomics Group—Centre for Ecology, Evolution and Environmental Changes (cE3c), Faculdade de Ciências, Universidade de Lisboa, Lisboa, Portugal; 5 Coffee Research Institute, Kenya Agricultural and Livestock Research Organization (KALRO), Ruiru, Kenya; 6 Centro Nacional de Investigaciones de Café (Cenicafé), Manizales, Colombia; Fujian Agriculture and Forestry University, CHINA

## Abstract

Understanding the molecular mechanisms underlying coffee-pathogen interactions are of key importance to aid disease resistance breeding efforts. In this work the expression of genes involved in salicylic acid (SA), jasmonic acid (JA) and ethylene (ET) pathways were studied in hypocotyls of two coffee varieties challenged with the hemibiotrophic fungus *Colletotrichum kahawae*, the causal agent of Coffee Berry Disease. Based on a cytological analysis, key time-points of the infection process were selected and qPCR was used to evaluate the expression of phytohormones biosynthesis, reception and responsive-related genes. The resistance to *C*. *kahawae* was characterized by restricted fungal growth associated with early accumulation of phenolic compounds in the cell walls and cytoplasmic contents, and deployment of hypersensitive reaction. Similar responses were detected in the susceptible variety, but in a significantly lower percentage of infection sites and with no apparent effect on disease development. Gene expression analysis suggests a more relevant involvement of JA and ET phytohormones than SA in this pathosystem. An earlier and stronger activation of the JA pathway observed in the resistant variety, when compared with the susceptible one, seems to be responsible for the successful activation of defense responses and inhibition of fungal growth. For the ET pathway, the down or non-regulation of ET receptors in the resistant variety, together with a moderate expression of the responsive-related gene *ERF1*, indicates that this phytohormone may be related with other functions besides the resistance response. However, in the susceptible variety, the stronger activation of *ERF1* gene at the beginning of the necrotrophic phase, suggests the involvement of ET in tissue senescence. As far as we know, this is the first attempt to unveil the role of phytohormones in coffee-*C*. *kahawae* interactions, thus contributing to deepen our understanding on the complex mechanisms of plant signaling and defense.

## Introduction

Coffee Berry Disease (CBD), caused by the hemibiotrophic fungus *Colletotrichum kahawae* J.M. Waller & P.D. Bridge, is a major constraint of Arabica coffee production in Africa. This disease may cause up to 50–80% of crop losses, in years of severe epidemics if control measures are not applied [1]. Since the first report in 1922 in Kenya [2], CBD is still restricted to Africa but there are major concerns about the risk of its introduction into Latin America and Asia [1,3]. *C*. *kahawae* infects several coffee organs, but major losses result from the infection of green berries. The outbreak of the disease with visible symptoms occurs during the expanding stage of berry development, producing black, sunken anthracnose-like lesions on the green pulp [4,5]. Although the application of fungicides can provide adequate control, the use of coffee resistant varieties is the most appropriate and sustainable management strategy against this disease.

Inheritance studies carried out in Kenya by van der Vossen and Walyaro (1980) [6] and recent molecular studies [7,8] provided evidences that coffee resistance to *C*. *kahawae* appears to be controlled by major genes in different loci. Cytological and biochemical studies revealed that coffee resistance to *C*. *kahawae* is characterized by restricted fungal growth associated with several host responses, such as hypersensitive-like cell death (HR), formation of cork barriers, callose deposition around intracellular hyphae, accumulation of phenolic compounds (flavonoids and hydroxycinnamic acid derivatives), lignification of host cell walls and increased activity of oxidative enzymes, such as peroxidases [3,9–13]. More recently, differentially expressed genes involved in recognition, signaling and defense responses of coffee to *C*. *kahawae* have been identified [14–16].

Plant growth and responses to environmental cues are largely governed by phytohormones. Recent research indicates that an antagonist/synergetic crosstalk among different phytohormones [particularly salicylic acid (SA), jasmonic acid (JA) and ethylene (ET)] play a central role in the regulation of plant immune responses to the pathogen [17,18]. These defense responses are considered to be dependent on the pathogen lifestyle and the genetic constitution of the host [19–22].

In plants, SA can be synthesized via two distinct enzymatic pathways that require the primary metabolite chorismate: phenylalanine ammonia-lyase (PAL)-mediated phenylalanine and isochorismate synthase (ICS)-mediated isochorismate. The NPR1 (non-expressor of pathogenesis-related genes 1) represents a key node in signaling downstream from SA (S1A Fig) [23]. In the absence of SA or pathogen challenge, NPR1 is inactive in cytoplasm as an oligomer. Upon induction, SA accumulation is promoted and NPR1 monomer is released to enter the nucleus where it activates defense gene transcription, such as the *PR1* gene [24].

JA biosynthesis starts with the release of α-linolenic acid (α-LA) from membrane lipids and its oxygenation in the chloroplast, followed by the sequential action of allene oxide synthase (AOS) and allene oxide cyclase (AOC), resulting in the synthesis of 12-oxophytodienoic acid (OPDA) (S1B Fig) [25,26]. OPDA migrates to the peroxisome to be reduced by oxophytodienoate redutase 3 (OPR3) and undergo several rounds of β-oxidation to form JA. Then, JA is exported from the peroxisome to cytosol for conjugation to the L-isoleucine (Ile) by jasmonate-resistant 1 (JAR1) resulting in the endogenous bioactive form of JA-Ile [27,28]. JA-Ile then interacts with coronatine insensitive 1 (COI1), an F-box protein which acts as a JA receptor, and targets jasmonate negative regulators [like jasmonate zim domain proteins (JAZ)] for degradation, promoting JA-induced gene transcription such as of *PR10* [25,29].

Ethylene is produced from methionine via S-adenosyl-L-methionine (SAM) being the last two biosynthetic steps catalyzed by aminocyclopropane-1-carboxylic synthase (ACS) and ACC oxidase (ACO) (S1C Fig) [30–32]. In the presence of ET, the receptor ethylene resistant 1 (ETR1) binds to the hormone switching off the constitutive triple response 1 (CTR1). Consequently, desphosphorylation of C-terminal end of ethylene insensitive 2 (EIN2) is promoted and the repression of the transcriptional signaling cascade is unlocked [33].

Despite the recent advances achieved on the complex regulation of phytohormones network in plants under different environmental conditions [34–36], this knowledge is still very scarce in plant-*Colletotrichum* spp. pathosystems, particularly in the coffee-*C*. *kahawae* interaction. The aim of this study was to elucidate the possible involvement of the phytohormones SA, JA and ET in the responses of coffee plants resistant (variety Catimor 88) and susceptible (variety Caturra) to *C*. *kahawae*. Based on a cytological analysis of the fungal growth and the associated host responses, time-points of the infection process were chosen to evaluate the expression of phytohormones biosynthesis, reception and responsive-related genes by quantitative real-time PCR (qPCR).

## Material and methods

### Plant material and inoculation

Experimental assays were conducted on coffee hypocotyls, since previous studies shown a correlation between the pre-selection test on hypocotyls and mature plant resistance in the field (r = 0.73–0.80) [37].

For this study, the varieties Catimor 88 (Timor hybrid derivative, which exhibit field resistance to *C*. *kahawae* breeding programmes in Kenya) and Caturra (CIFC 19/1 –*Coffea arabica* L.) were used as resistant and susceptible varieties to *C*. *kahawae* isolate Que2 (from Kenya), respectively. Coffee seeds were sown in a mixture of soil:peat:sand (1:1:1) and grown under greenhouse conditions with average temperatures between 16°C and 28°C (minimum and maximum temperatures, respectively) during 8 weeks.

Conidia of *C*. *kahawae* isolate Que2, retrieved from a *C*. *kahawae*, collection maintained at CIFC/ISA were produced after 7 days at 22°C on malt extract agar (MEA) [38]. Hypocotyls of Catimor 88 and Caturra, were inoculated according to the technique described by van der Vossen *et al*., (1976) [37] with slight modifications. Briefly, hypocotyls were vertically placed on plastic trays containing a wet nylon sponge and sprayed with a conidia suspension (3x10^6^/ml). The trays were then covered with a plastic bag to simulate a humid chamber and were kept in a Phytotron750 E at 22°C. For the first 24 hours post inoculation (hpi), trays were kept in the dark and afterwards a 12 hours photoperiod was established for the remaining time-course of the experiment. Non-inoculated hypocotyls sprayed with water were kept in the same conditions as the inoculated hypocotyls and used as control.

### Light microscopic observation in fresh tissues

Conidial germination and appressorial differentiation were observed on hypocotyl pieces (5cm^2^) at 3, 6, 9, 12, 15, 18 and 24hpi, as previously described [39]. The hypocotyl pieces were painted with transparent nail polish on the inoculated surface to recreate a tissue surface replica. Once dried, the nail polish was removed, stained and mounted in lactophenol cotton blue. For each experiment, a minimum of six microscope fields, each containing 100 conidia and/or differentiated appressoria on the surface of hypocotyls, were used. To evaluate fungal post-penetration growth stages at 24, 48 and 72hpi, cross sections of inoculated hypocotyl fragments made with a freezing microtome (Leica CM1850), were stained and mounted in lactophenol cotton blue [11,39]. Hyphal length inside hypocotyl tissues were estimated with the aid of a micrometric eyepiece.

To detect autofluorescent cells, cross sections of non-inoculated (control) and inoculated tissues were placed in 0.07M pH 8.9 phosphate solution (K_2_HPO_4_) for 5 min, and mounted in the same solution [11,40]. Autofluorescence under epifluorescence blue light is thought to indicate the presence of phenolic-like compounds and cytoplasmic autofluorescence and/or browning is frequently associated with plant cell death [41,42]. Observations were made using light microscopes (LeitzDialux 20 and Leica DM-2500) equipped with a mercury bulb HB 100W blue light (excitation filter BP 450 and 490; barrier filter LP 515).

Data on pre- and post-penetration fungal growth stages and host cell responses are presented as the combined values of three experiments. Fungal growth inside host tissues and plant responses were recorded from 75 to 100 infection sites per experiment at three time-points (24, 48 and 72hpi).

### RNA extraction and cDNA synthesis

Hypocotyls (inoculated and non-inoculated) were harvested at 6, 12, 24, 48 and 72hpi, immediately frozen in liquid nitrogen and stored at -80°C prior to RNA extraction. Three biological replicates, each representing a pool of 15 hypocotyls, were used.

Total RNA was extracted with the Spectrum Plant Total RNA kit (Sigma-Aldrich, USA), according to the manufacturer’s instructions. Residual genomic DNA was digested with DNase I (On-columm DNase I Digestion Set, Sigma-Aldrich, USA). RNA purity and concentration was measured at 260/280 nm and 260/230 nm using a spectrophotometer (NanoDrop 1000, Thermo Scientific). RNA integrity was verified by electrophoresis in 1% agarose gel. Genomic DNA (gDNA) contamination was checked by qPCR analysis on the crude RNA [43]. Complementary DNA (cDNA) was synthesized from 2.5μg of total RNA using RevertAid^®^H Minus Reverse Transcriptase (Fermentas, Ontario, Canada) anchored with Oligo(dT) 23primer (Fermentas, Ontario, Canada), according to manufacturer’s instructions.

### Primer design

Within the targeted phytohormone pathways, sixteen genes related with biosynthesis, reception and responsiveness [25,33,44] were selected for expression analysis in *Coffea* spp. after *C*. *kahawae* challenge (Table 1). For SA, the following genes were included: Isochlorismate synthase 2 (*ICS2*); Phenylalanine ammonia-lyase (*PAL)*; Non-expressor of pathogenesis-related 1 (*NPR1)*; Pathogenesis-related (*PR1*). For JA the following genes were included: 12-oxoplytodienotae reductase 1-like (*OPR3)*; Coronatine insensitive 1 (*COI1)*; Pathogenesis-related 10 (*PR10*). For ET the following genes were included: 1-aminocyclepropane-1-carboxylic acid synthase 5 (*ACS5)*; 1-aminocyclepropane-1-carboxylic acid oxidase 2 (*ACO2)*; Ethylene resistant 1 (*ETR1)*; Ethylene insensitive 2 (*EIN2)*; Constitutive triple response 1 (*CTR1)*; Ethylene-responsive factor 1 (*ERF1*). With the exception of *PAL* and *PR10* genes, that were previously described in coffee [45], the remaining fourteen genes were retrieved from a coffee RNA-seq database [46] as being orthologous of previously described genes in *Arabidopsis thaliana* (TAIR database:www.arabidopsis.org). Tubulin beta-9 (*β-Tub9*)/ ribosomal protein S24 (*S24)* and Insuline Degrading Enzyme (*IDE*)/*S24* were used as reference genes for susceptible and resistant varieties samples, respectively [14]. Coffee specific primers (Table 1) were designed with PrimerSelect version 5.0 (DNAStar, Inc., USA) using the following parameters: amplicon length 70 and 200 bp; size between 17 and 22 bp; annealing temperature (Ta) between 58 and 62°C and GC content± 50%.

**Table 1:** Primer sequences for qPCR analysis of target and reference genes.

Gene	Coffee source name	Name	Primer sequence (5'-3')	Primer length (bp)	Amplicon length (bp)	Ta (°C)	Tm (°C)	PCR efficiency
*ICS2*	Scaffold21359	Isochorismate synthase 2	Fw: TGCCATAGTACGAGAAAACA	20	124	60	79.0	94
			Rev: CCCAGAAAATCGACCATAAA	20				
*PAL*^a^	CaPAL F 13097	Phenylalanine ammonia-lyase	Fw: GCAGGTCCTACTCATTGTACAAG	23	166	60	82.0	89
			Rev: CCATTCCACTCTTTCAAACAATCC	24				
*NPR1*	Scaffold33187	Non-expressor of PR1	Fw: AGGGCATTGGATTCTGACGA	20	126	60	81.5	88
			Rev: CTCTGTTGTGGTCTTTGCGT	20				
*PR1*	Scaffold170607	Pathogenesis-related 1	Fw: GCCCGTAAAGTCACCTGT	18	177	60	86.5	91
			Rev: AACTACGCTGCCAAAATC	18				
*OPR3*	Scaffold2739	12-oxoplytodienotae reductase 1-like	Fw: ATAACTCCCCACCTTCCAAC	20	198	58	81.5	91
			Rev: ACAGCCTTATCCCAACTCTAT	22				
*COI1*	Scaffold40077	Coronatine insensitive 1	Fw: CTTAGCATCACCACCCACC	19	157	62	81.5	93
			Rev: TCCGATCCCCCATACCAAC	19				
*PR10*^b^	CF589103	Pathogenesis-related 10	Fw: GCCACCATCCTTGAAGAGAA	20	151	55	80.0	99
			Rev: CAACTCTCTGCTTGGCAGTCT	21				
*ACS5*	Scaffold82864	1-aminocyclepropane-1-carboxylic	Fw: AGGGCGTCCTGGTCACTAA	19	148	60	83.0	90
		acid synthase 5	Rev: CTCGGCGAGCTAAAAACTGT	20				
*ACO2*	Scaffold57328	1-aminocyclepropane-1-carboxylic	Fw: AAAGTCAGCAATTACCCTCCA	21	144	58	87.0	93
		acid oxidase 2	Rev: ATCCACCCATTCACCATCCT	20				
*ETR1*	Scaffold776	Ethylene resistant 1	Fw: GCCCCCAAGATATTCCTAAG	20	92	60	79.5	89
			Rev: TGCAAGACCAAGACCACTAC	20				
*EIN2*	Scaffold3828	Ethylene insensitive 2	Fw: GTTACTTCTCCAAAACCTACT	21	134	60	78.5	93
			Rev: TCCCATTTACCACTCTTATCT	21				
*CTR1*	Scaffold16054	Constitutive triple response 1	Fw: GCAGCTGTGGGTTTCAAGG	19	162	60	83.0	90
			Rev: AGTGGGGGAGGGTTTAGTC	19				
*ERF1*	C312112	Ethylene-responsive factor 1	Fw: TGGCTGGGCACATTTGAC	18	85	58	84.0	90
			Rev: GGATTGCTGCTTGACCTC	18				
*IDE*^c^	isotig10635	Insuline Degrading Enzyme	Fw: TGATCTAAGCTGGTGGAAAGC	21	91	55	76.3	99
			Rev: TCAGGTGCATCAGGATGATT	20				
*S24*^c^	SNG-U349723*	Ribosomal protein S24	Fw: GCCCAAATATCGGCTTATCA	20	92	60	7.6	95
			Rev: TCTTCTTGGCCCTGTTCTTC	20				
*β-Tub9*^c^	isotig08544	Tubulin beta-9	Fw: ACCCTCCAGCAAACTGATGA	20	100	55	77.3	92
			Rev: AGGATGCCACTGCTGATGAT	20				

bp–base pairs, Ta–Annealing temperature, Tm–Melting temperature

* Unigene accession number according to the SOL Genomics Network.

^a^ Fernandez *et al*., (unpublished).

^b^ Ramiro *et al*., 2009 [45].

^c^ Figueiredo *et al*., 2013 [14].

### Quantitative real-time PCR

The qPCR experiments were carried out using SYBR Green Supermix (BioRad) in an iQ5 real-time thermal cycler (Bio-Rad, USA). Each 25μl reaction comprised 4μl cDNA template (2.5μg/μl), 12.5μl SYBR Green Supermix (Bio-Rad, USA), 0.4μl of each primer (10μM) and 0.7μl of sterile distilled water. Thermal cycling started with a denaturation step at 95°C for 10 min, followed by 45 cycles of denaturation at 95°C for 15s and annealing at the respective temperature for each gene (Table 1) for 30s. Each set of reactions included a negative control with no template. The amplification efficiency for each gene of interest was determined using the LinRegPCR version 2013.0. Dissociation curves (S2 Fig) and agarose gel electrophoresis were used to analyze non-specific PCR products. Three biological replicates and two technical replicates were used for each sample. Relative gene expression (fold change) was calculated according to Hellemans *et al*., (2007) [47]. The gene expression data were further visualized using the software MeV viewer (http://www.tm4.org).

### Statistical analysis

For statistical analysis of cytological data, Student’s *t* was applied using IBM^®^SPSS^®^ Statistics version 20.0 (SPSS Inc., USA) software and arcsine-transformed percentages were used where appropriate. Statistical significance (p≤0.05) of gene expression between the two coffee varieties was determined by the non-parametric Mann–Whitney *U* test using IBM^®^SPSS^®^ Statistics version 20.0 (SPSS Inc., USA) software.

## Results

### Fungal infection and host responses

**Fig 1:**
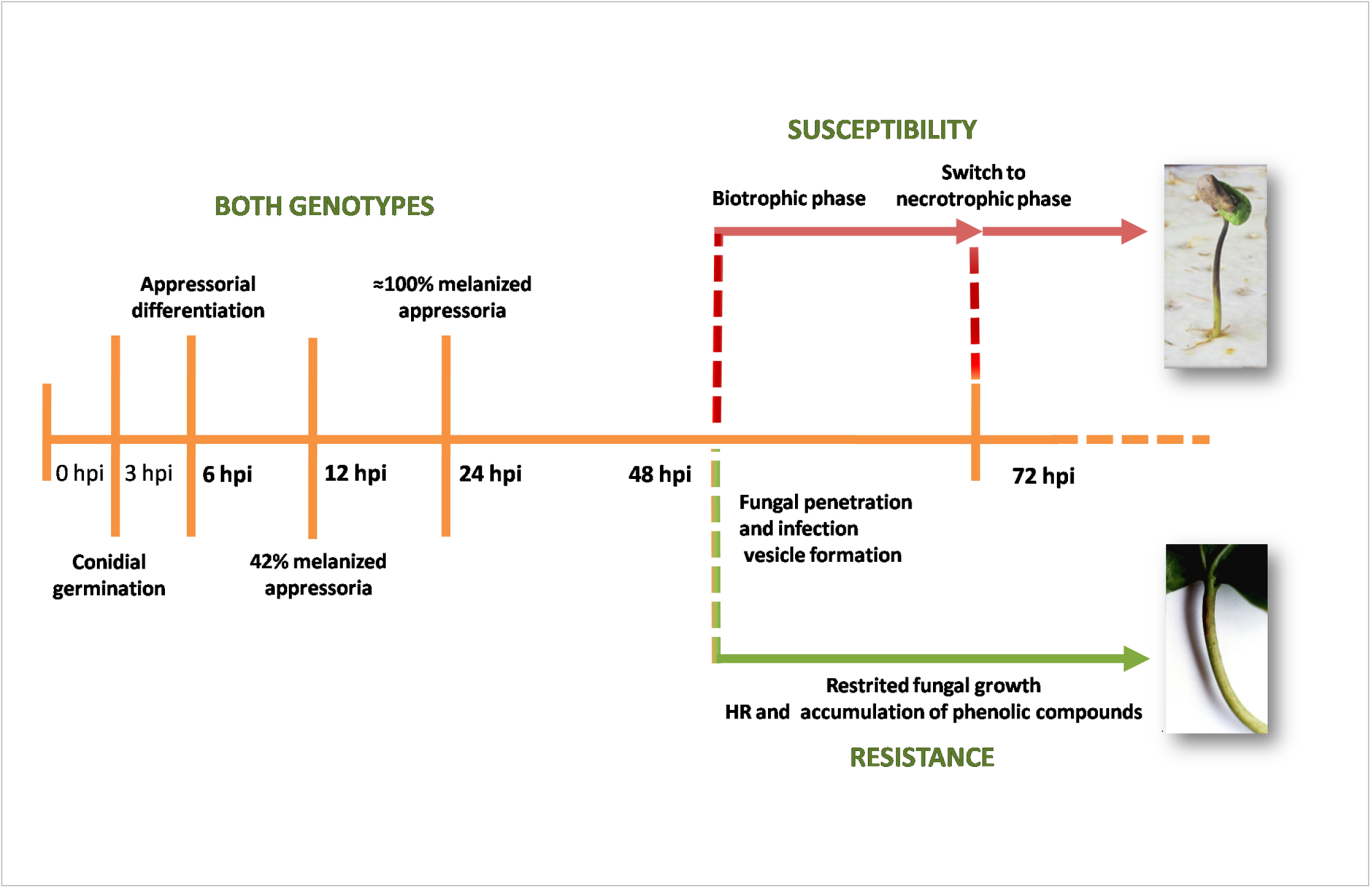
Time-course of *Colletotrichum kahawae* (isolate Que2) infection in hypocotyls of resistant (Catimor 88) and susceptible (Caturra) coffee varieties. **3hpi and 6hpi**–beginning of conidia germination and appressoria differentiation, respectively; **12hpi**– 42% of melanized appressoria; **24hpi**–almost 100% of melanized appressoria; **48hpi**—fungal penetration and biotrophic growth; **72hpi**–switch to necrotrophy (susceptible variety) and restriction of fungal growth (resistant variety). Times after inoculation (bold) were selected to collect samples for gene expression studies by qPCR.

**Fig 2:**
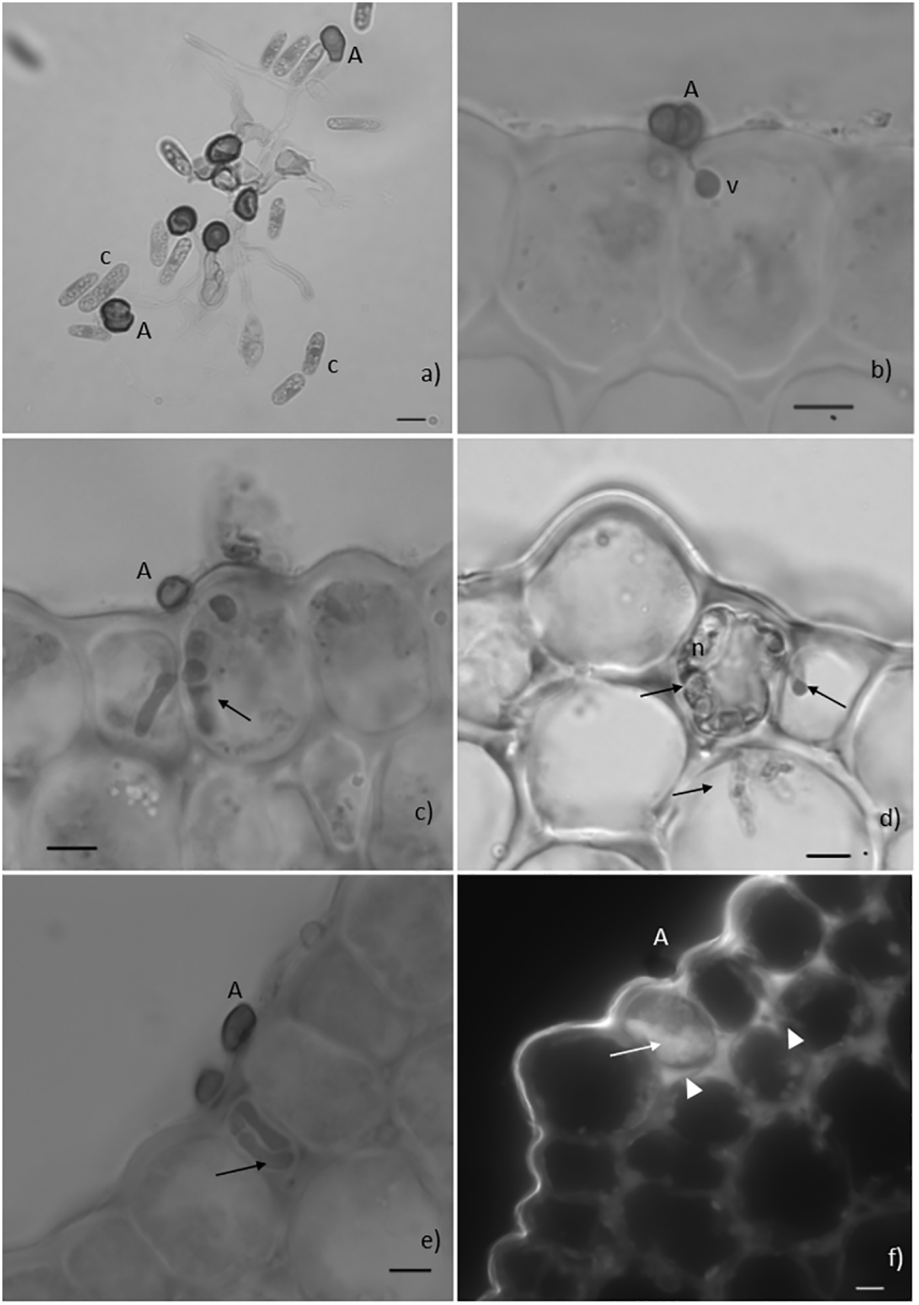
Fungal pre-and post-penetration growth stages and host responses. Light microscope observations, cotton blue lactophenol staining (2a-2e) and epifluorescence test under blue light (2f). **Fig 2a.** Conidia (c) germination and formation of melanized appressoria (A) on the surface of a resistant hypocotyl, 24 hours post inoculation (hpi). **Fig 2b.** Infection site showing a melanized appressorium (A) and an infection vesicle (v) in the epidermal cell of the resistant hypocothyl, 48hpi. **Fig 2c.** Infection site showing a melanized appressorium (A) and hyphae inside two adjacent epidermal cells of the susceptible hypocotyl (arrow), 48hpi. **Fig 2d.** Fungal hyphae (arrows) in living and in necrotized (n) cells of the susceptible hypocotyl, 72hpi. **Fig 2e.** Infection site showing a melanized appressorium (A) and intracellular hyphae (arrow) confined to the epidermal cell of the resistant hypocotyl, 72hpi. **Fig 2f.** Infection site showing an appressorium (A) associated with autofluorescence of the cytoplasmic content of one epidermal cell (HR-like). Note that the walls of this cell and of adjacent epidermal and cortex cells are also autofluorescent (bars = 10μm).

The development of pre-penetration fungal growth stages of *C*. *kahawae* was similar in both resistant and susceptible varieties (Fig 1 and Table 2). Conidial germination and appressorial differentiation were initiated at 3 and 6hpi, respectively. At 12hpi, 42% of the appressoria were melanized reaching 89–94% at 24hpi (Fig 2A). In both coffee varieties, the melanized appressoria began to penetrate the epidermal cells with the formation of a globose infection vesicle, at 48hpi (Fig 2B). Hyphae developed from the vesicles grew either intra- and intercellularly colonizing epidermal and cortex cells. In the susceptible variety, the fungus pursued its growth feeding on living host cells (biotrophy) before switching to necrotrophy (72hpi) in the majority of the infection sites (about 90%) (Fig 2C and 2D). Resistance was characterized by restricted fungal growth (fungal hyphae were more frequently confined to the epidermal cells or occasionally to those of the first layer of the cortex cells), as confirmed by the evaluation of hyphal length (Table 3) (Fig 2E and 2F). The entanglement of fungal hyphae originating from different infection sites, together with the increasing number of necrotic host cells in susceptible tissues, did not allow the quantification of the hyphal length beyond 72hpi.

**Table 2:** Percentage of conidial germination and appressorial differentiation of *Colletotrichum kahawae* on hypocotyls of resistant and susceptible coffee varieties, at different hours post inoculation.

Hours post inoculation	Coffee varieties	Germinated conidia (%) (x ± SD)	*t* test*	Appressoria** (%) (x ± SD)	*t* test*	Melanized appressoria (%) (x ± SD)	*t* test*
**3 h**	Catimor 88 (R)	3±3	0.15 ^ns^	0	-	0	-
	Caturra (S)	4±4		0		0	
**6h**	Catimor 88 (R)	16±13	0.90^ns^	43±26	0,32^ns^	1±1	0.37^ns^
	Caturra (S)	16±33		46±33		2±2	
**9h**	Catimor 88 (R)	26±9	0.35^ns^	65±16	0.92^ns^	17±12	0.06^ns^
	Caturra (S)	29±15		65±36		9±9	
**12h**	Catimor 88 (R)	36±33	0.32^ns^	69±18	0.34^ns^	42±11	0.92^ns^
	Caturra (S)	33±26		76±27		42±31	
**15h**	Catimor 88 (R)	50±18	0.34^ns^	78±11	0.94^ns^	49±17	0.34^ns^
	Caturra (S)	57±17		79±8		56±26	
**18h**	Catimor 88 (R)	61±18	0.36^ns^	87±13	0.95^ns^	72±20	0.95^ns^
	Caturra (S)	65±24		88±10		71±23	
**24h**	Catimor 88 (R)	71±22	0.96^ns^	89±14	1,16^ns^	89±11	1.16^ns^
	Caturra (S)	72±20		94±6		94±6	

R = Resistant; S = Susceptible; X ± SD = mean ± standard deviation

* Student’s *t* test (ns—non significative)

** Total of appressoria (nonmelanized and melanized)

**Table 3:** Evaluation of fungal growth in coffee hypocotyls of resistant and susceptible coffee varieties after challenge with *Colletotrichum kahawae*, at different hours post inoculation.

	Hyphal length (μm)/infection site in hypocotyls of coffee varieties		
Hours post inoculation	Catimor 88 (R) x± SD	Caturra (S) x± SD	*t* test*
24	0	0	_
48	3.94±2.11	8.43±8.22	1.49^ns^
72	12.76±8.81	42.6±32.52	4.25***

R = Resistant; S = Susceptible; X ± SD, mean ± standard deviation

* Student’s *t*-test (ns—non significative

***p≤0.001)

**Fig 3:**
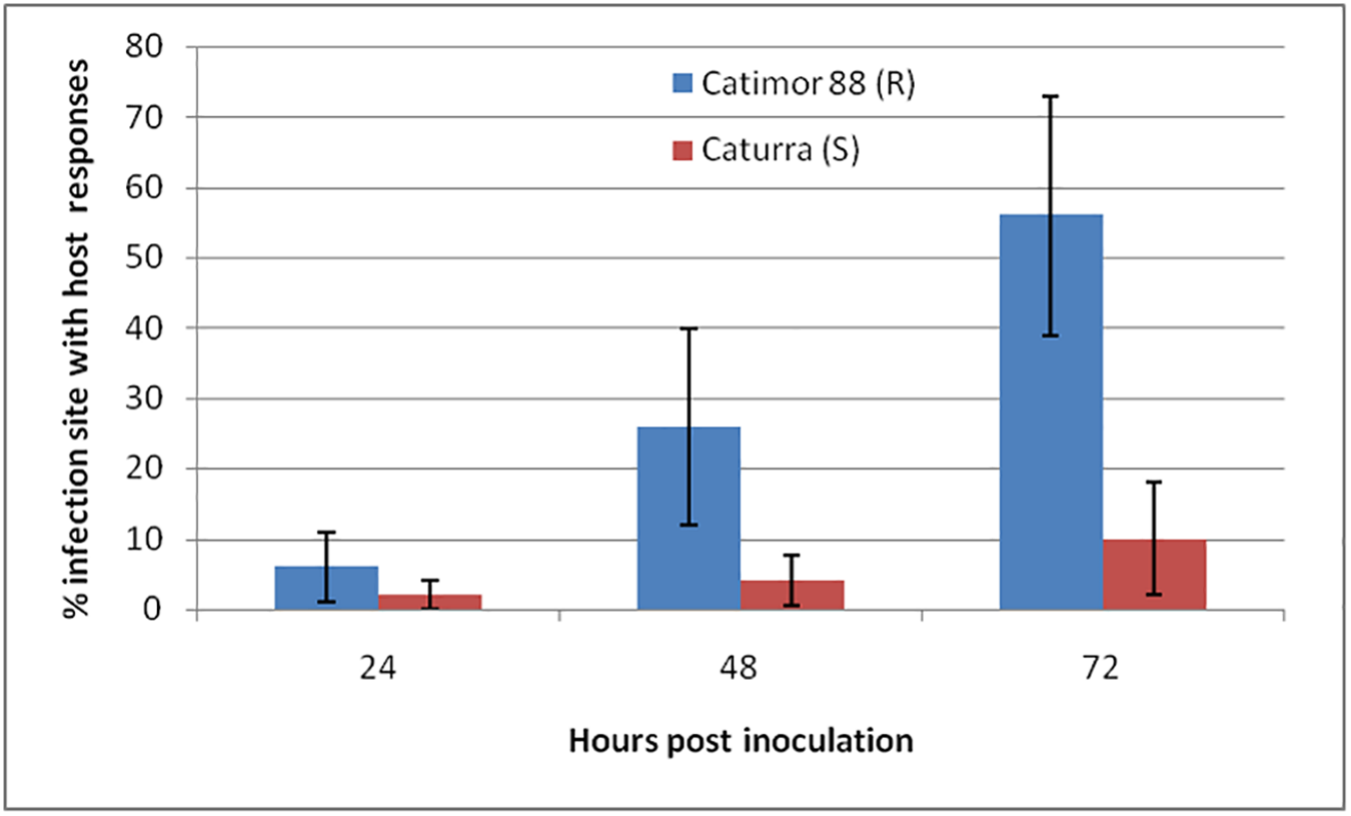
Percentage of infection sites with host responses. Accumulation of phenolic-like compounds in the cell walls only, or in both the cell walls and the cytoplasmic contents and deployment of hypersensitive-like cell death (HR) induced by *C*. *kahawae* in hypocotyls of coffee varieties Catimor 88 (R-resistant) and Caturra (S-susceptible), at different hours post inoculation. The average percentages were significantly higher in the resistant than in the susceptible coffee variety at 24hpi (*t* = 2.52; p ≤ 0.05), 48hpi (*t* = 4.83; p ≤ 0.001) and 72hpi (*t* = 6.69; p ≤ 0.001).

**Fig 4:**
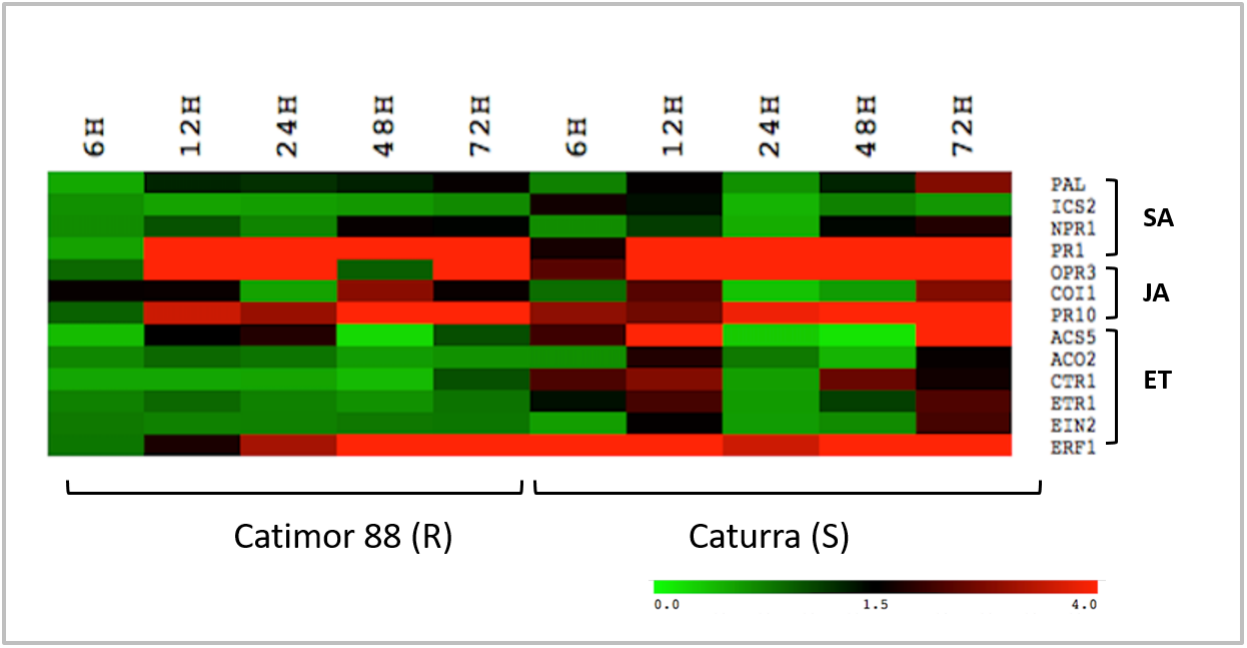
Expression analysis of SA, JA and ET pathway related genes in non-inoculated hypocotyls of Catimor 88 (R-resistant) and Caturra (S-susceptible) vs inoculated hypocotyls with *C*. *kahawae*. Heatmap was colored according to the log2 ratio of expression, where green indicates lower expression, red indicates higher expression and black indicates no expression (see the color scale); in columns are the time-point studied (6, 12, 24, 48 and 72hpi) and in the rows the genes analyzed.

In the resistant variety, the first cytological changes were displayed in the epidermal cells at 24hpi and corresponded to: (i) accumulation of phenolic-like compounds [indicated by autofluorescence (AF) in cell walls only or in cell walls plus the cytoplasmic content]; (ii) deployment of HR (monitored by the AF and/or browning of the cytoplasmic contents) (Figs 2F and 3). During the time course of the infection, these responses spread to adjacent cells of the epidermis and of the first layer of cortex cells. Similar responses were detected in the susceptible variety, but in a significantly lower percentage of infection sites (24hpi: 2%, 48hpi: 4%, 72hpi: 10%) comparatively to the resistant variety (24hpi: 6%, 48hpi: 26%, 72hpi: 56%) (Fig 4). In these infection sites the fungus stopped its growth at the stage of appressoria or infection vesicle.

At 5–6 days after inoculation, resistant hypocotyls exhibited scab lesions or, occasionally absence of macroscopic symptoms, whereas susceptible hypocotyls showed typical dark sunken lesions with sporulation (Fig 1).

### Expression of genes from SA, JA and ET pathways

**Fig 5:**
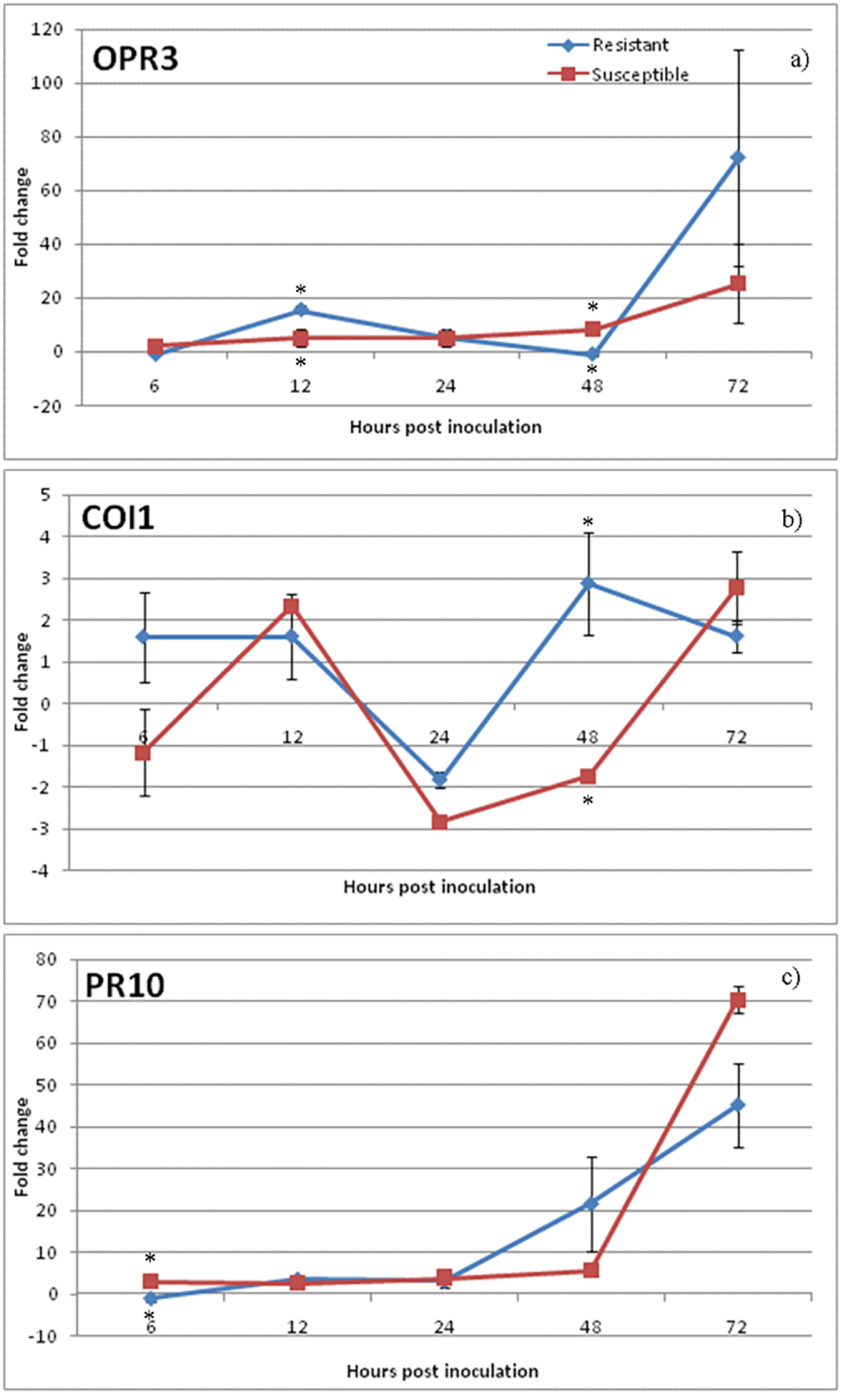
qPCR expression analysis of JA pathway related genes. Relative expression patterns of *OPR3* (biosynthesis), *COI1* (receptor) and *PR10* (responsive gene) obtained in Catimor 88 (R-resistant) and Caturra (S-susceptible) coffee varieties. Mean and standard deviation of three biological replicates is presented. Fold change as relative expression of gene expression between inoculated and control samples for each of the coffee varieties/inoculation time-points. Asterisks (∗) represent statistical significance (p≤0.05) of gene expression between the two coffee varieties was determined by the non-parametric Mann–Whitney *U* test using IBM^®^SPSS^®^ Statistics version 20.0 (SPSS Inc., USA) software.

Phytohormone pathway induction during *Coffea* spp.-*C*. *kahawae* interaction was monitored by the expression analysis of sixteen related genes. For a global perspective of the relative expression levels of SA, JA and ET pathway-related genes in the resistant and susceptible varieties along the infection process, a heatmap analysis was performed (Fig 4). The expression patterns of SA pathway-related genes were quite similar for both coffee varieties; being SA biosynthesis and receptor-related genes (*ICS2*, *PAL*, *NPR1*) mostly non-regulated, while *PR1* gene was up-regulated from 12hpi onwards (Fig 4 and S3 Fig). On the contrary, the expression level of genes from JA and ET pathways showed differences between the two varieties (Fig 4). The activation of the JA pathway was observed in both coffee varieties, but differently in timing and magnitude (Fig 5). In the susceptible variety, the biosynthesis-related gene *OPR3* was up-regulated in all time-points showing the highest level of expression at 72hpi (25.5±14.9). JA-Ile receptor gene *COI1* was up-regulated at 12hpi (2.3±0.14) and 72hpi (2.7±0.9), while the responsive–related gene *PR10* showed the maximum value of expression at 72hpi (70.3±3.3). In the resistant variety, the up-regulation of the biosynthesis-related gene *OPR3* was observed at 12-24hpi (12hpi: 15.5±1.4; 24hpi: 5.5± 3.3) and later at 72hpi (72.2±40.4). The maximum of expression of the JA-Ile receptor gene *COI1* was observed at 48hpi (2.8±1.2) which was coincident with the strong increase in expression level of the responsive–related gene *PR10* from 48hpi onwards (48hpi: 21.5±11.3; 72hpi:45.2±10.1).

**Fig 6:**
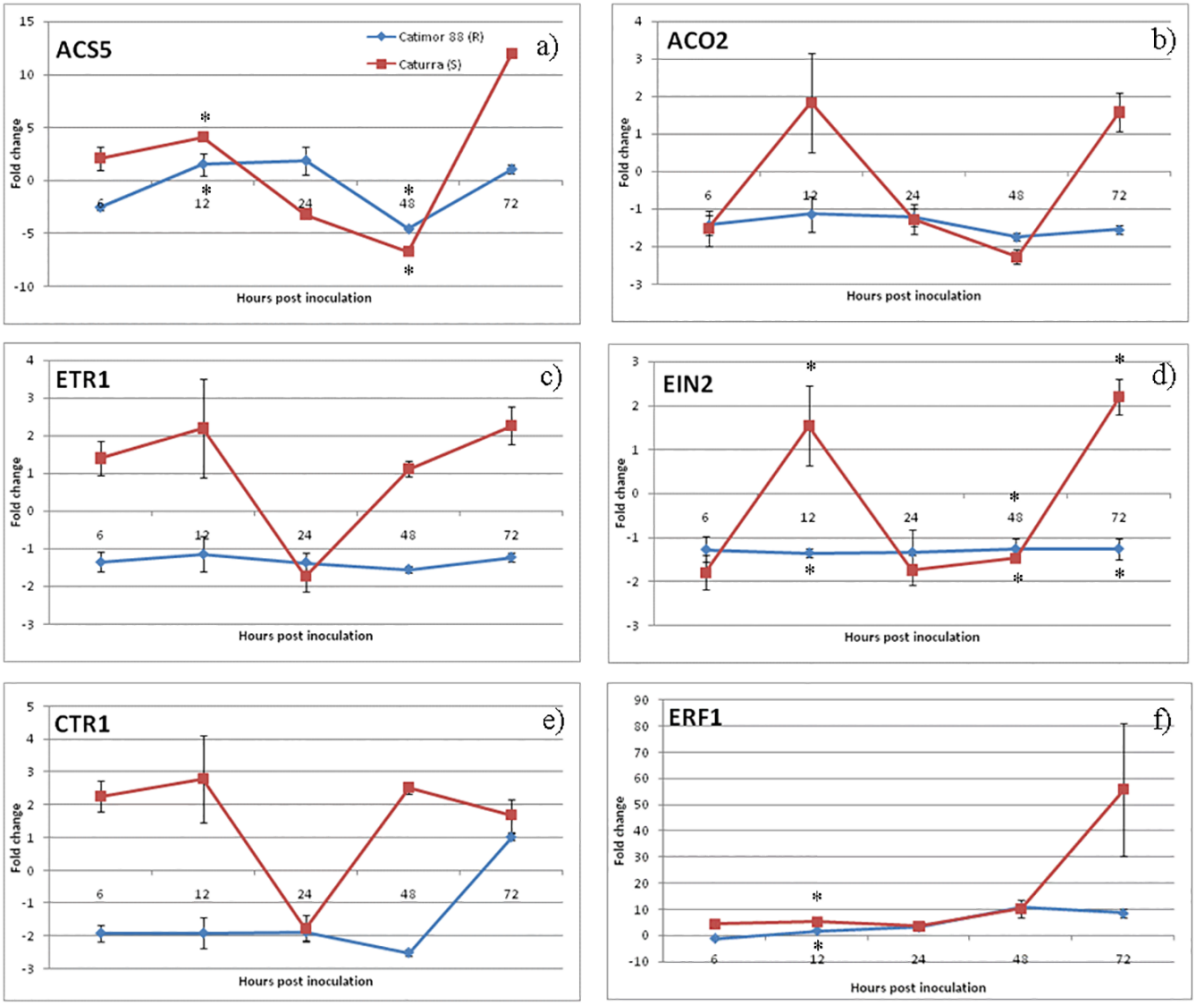
qPCR expression analysis of ET pathway related genes. Relative expression patterns of *ACS5/ACSO2* (biosynthesis), *ETR1/EIN2* (receptors), *CTR1* (negative regulator) and *ERF1* (responsive gene) were obtained in Catimor 88 (R- resistant) and Caturra (S-susceptible) coffee varieties. Mean and standard deviation of three biological replicates is presented. Fold change as relative expression of gene expression between inoculated and control samples for each of the coffee varieties/inoculation time-points. Asterisks (∗) represent statistical significance (p≤0.05) of gene expression between the two coffee varieties was determined by the non-parametric Mann–Whitney *U* test using IBM^®^SPSS^®^ Statistics version 20.0 (SPSS Inc., USA) software.

An activation of the ET pathway was also observed in both varieties but with differences in the ET-receptor genes expression profiles (Fig 6). In the susceptible variety, the biosynthesis-related genes *ACS5* and *ACO2* presented similar expression profiles being both up-regulated at 12hpi (*ACS5*: 4.1±0.2; *ACO2*: 1.8±1.3) and 72hpi (*ACS5*: 12±0.3; *ACO2*: 1.6±0.5) and down-regulated at 24hpi and 48hpi. ET-receptor gene *ETR1* and central ET signaling regulator *EIN2*, together with the negative ET pathway regulator *CTR1* showed similar expression profiles being all up-regulated at 12hpi, and down-regulated at 24hpi followed by an up-regulation at 72hai. ET-responsive gene *ERF1* was moderately activated from 6hpi to 48hpi, reaching a maximum value of expression at 72hpi (55.8±25.5). In the resistant variety, the biosynthesis-related gene *ACS5* was up-regulated at 12-24hpi (12hpi; 1.5±1.1; 24hpi: 1.8±1.3) while *ETR1*, *EIN2* and *CTR1* were down or non-regulated along the infection process. ET-responsive gene *ERF1* was moderately activated at all time-points, with an increase in expression level at 48-72hpi (48hpi: 10.7±1.0; 72hpi: 8.5±1.6).

## Discussion

In this work, the involvement of phytohormones in the deployment of coffee susceptible and resistance response to *C*. *kahawae* was studied. In the two coffee varieties, no differences were observed in fungal development from conidia germination to differentiation of melanized appressoria and fungal penetration. In the majority of the infection sites of the susceptible variety, the fungus pursued its growth without apparent inhibition, first establishing a biotrophic interaction with the host cells and later (at 72pai) switching to a destructive necrotrophic phase, as described for many species of *Colletotrichum* [48–51]. Conversely, as previously described by Silva *et al*., (2006) [3] and Loureiro *et al*., (2012) [11], in the resistant variety the restricted hyphal growth was associated with the hypersensitive-like host cell death (HR), and early accumulation of phenolic compounds both in cell walls and in the cytoplasmic contents. These responses were also observed in the susceptible variety, but in a significantly lower percentage of infection sites and did not prevent the fungal growth, as indicated by the appearance of typical anthracnose symptoms and the presence of acervuli. Time-course experiments carried out by Vargas *et al*., (2012) [52] also revealed that during the biotrophic growth in susceptible maize leaves, the hemibiotrophic fungus *C*. *graminicola* induced classical plant defense responses, such as the accumulation of reactive oxygen species and phenolic compounds. They hypothesized that it is the switch to necrotrophy that enables the fungus to evade the plant immune system and allows pathogenicity.

The classic view on the involvement of phytohormones in plant-pathogen interactions suggests that i) biotrophy is controlled by SA, while necrotrophy/hemibiotrophy is controlled by JA/ET, and that ii) SA is an antagonist of JA /ET [18]. Recent studies challenge these concepts revealing a very complex phytohormone self-regulation and cross-talk [25,53–58]. Indeed, in some reports on plant-hemibiotrophic pathogen interactions, SA signaling seems to be relevant for the outcome of resistance [53,59–63] while in others the role of SA is less clear [64,65]. In the coffee-*C*. *kahawae* interactions studied, the low expression of SA biosynthesis and receptor-related genes (*ICS2*/*PAL* and *NPR1*) turns also unclear the role of this phytohormone, although in coffee resistance against the biotrophic fungus *Hemileia vastatrix* a SA-dependent pathway seems to be involved [66,67]. The hormone-responsive gene *PR1* (commonly used as a SA pathway-marker) was unexpectedly induced in both susceptible and resistant coffee varieties when challenged with *C*. *kahawae*, but in a greater magnitude in the resistant variety. The apparent lack of coordination between the *PR1* expression profile and the other selected SA-pathway markers under study (*ICS2*, *PAL* and *NPR1*) suggests that in this pathosystem, *PR1* induction could be mediated by other phytohormones than SA. In accordance, the knockout of *OsPAL06I* in rice challenged with *Magnaporthe oryzae* did not influence the expression of *PR1a* compared to wild-type, although the SA and JA levels were significantly reduced in roots [53]. Tang *et al*., (2010) [68] reported a differential expression of *PR1* and chitinase genes in bananas matured and treated with ethephon (analog to ethylene), demonstrating that benzothiadizole (BTH–analog to SA) failed to induce them. Furthermore, an up-regulation of PR1, among others PRs, was observed after MeJA (methyl jasmonate) treatment [54, 69].

The same authors related the increase of PRs expression with the increase of disease resistance, and identified a high degree of consistency between the concentration fluctuation of endogenous JAs and the expression of PRs, therefore suggesting that accumulation of JAs may be one inner factor for regulation of PRs.

Our results also revealed an induction of the JA pathway in both varieties, however it occurred earlier and/or in a greater magnitude in the resistant than in the susceptible variety. The biosynthesis-related gene *OPR3* was induced in both varieties during the appressorial melanization (12-24hpi) and the beginning of host cell responses (24pai). Furthermore, an increase of the expression of JA-responsive gene *PR10* was observed at the beginning of fungal penetration (48pai) in the resistant variety, while in the susceptible variety it was coincident with the switch to necrotrophic fungal growth (72pai). In a similar way, Ding *et al*., (2011) [70] observed that, when wheat was challenged with the hemibiotrophic fungus *Fusarium graminearum*, only the variety with a high level of resistance, when compared with a susceptible mutant, had an increase in *OPR3* activation, concluding that the induction of JA pathway was involved in plant defense. Figueiredo *et al*., (2015) [25] also observed a significant increase of *OPR3* and *COI1* expression in the resistant grapevine (*Vitis vinifera*) cultivar “Regent” at the first hours post inoculation with the biotrophic fungus *Plasmopora viticola*, when compared with the susceptible cultivar “Trincadeira”, concluding that the timing of JA pathway induction was responsible for a set of efficient defense responses.

In our study, the earlier increase in *PR10* expression observed in the resistant variety as early as the fungal penetration stage, may reflect an early attempt of the coffee plant to halt the pathogen development, as is observed in other plant-pathogen interactions [54,71,72]. Although the precise function of PR10 proteins is poorly understood, some exhibit antimicrobial activity, and DNase and/or RNase activity [73,74]. Recent studies also suggest that PR10 may have an important role in the control of phenylpropanoid and flavonoid biosynthesis and their transport to sites where they are needed, such as the reinforcement of the cell wall [75]. This seems to be in line with the accumulation of phenolic-like compounds in the host cell walls found in our study.

The ET pathway seems also to be induced in both coffee varieties however with a remarkable difference in the ET-receptor expression profile, which may reflect the complexity of the role and regulation of this hormone. The ET pathway induction may start at the earliest hours post inoculation, since at least one of the two ET biosynthesis enzymes were found to be up-regulated during the appressorial melanization (12-24hpi) and at the onset of host cell responses (24hpi) in both coffee varieties. It was also observed that at the switch to the nectrophic phase (72hpi), an up-regulation of both ET biosynthesis enzymes occurred but only in the susceptible variety. However, it is not clear which enzyme, ACO or ACS, is the rate-limiting factor in ET biosynthesis. Different reports have suggested that either ACO activity is the key step for controlling ethylene production [76,77] or ACS is the rate-limiting enzyme [78]. In susceptible *Nicotiana benthamiana* challenged with *C*. *orbiculare*, an induction of *ACO* gene was observed during the biotrophic phase, with a maximum expression coincident with the switch to necrotrophic phase after which expression started to decrease. However, in NbACO1-silenced plants inoculated with *C*. *orbiculare*, a higher number of leaf lesions appeared earlier when compared with control plants, suggesting that ET might have some role in plant defense although it was not sufficient to stop the disease progress in this interaction [79]. High induction of *ACS* and *ACO* genes was also reported in susceptible citrus flowers inoculated with *C*. *acutatum* with a continued increase of expression of both genes up to 7 days after inoculation [80,81]. In this case, it was suggested that the induction of ET biosynthesis, and consequently the activation of ET pathway in susceptibility, was related with senescence of the tissues and promotion of young fruits drops, a characteristic of the Postbloom Fruit Drop disease [80,81]. The regulation of ET-receptors plays a major role in the ET signaling pathway. Ethylene induces receptor degradation through the 26S proteasome at the same time that transcriptional activation of new receptors is promoted. By this way, newly synthesized receptors not yet bonded with ethylene will allow a downstream pathway inhibition as soon as the levels of the hormone decreases [33]. In the susceptible coffee variety, the up/down-regulation profile of *ETR1* suggests that also in this pathosystem, ET-receptors may undergo a similar regulation process. However, in the resistant variety, the repression of *ETR1* throughout the infection process may suggest a different receptor regulation or even an alternative role for ET. In fact, the ET receptors are largely redundant in the control of ethylene responses but some functional specificity among their different isoforms has recently been uncovered [82], together with evidences of a degree of complexity of ET receptors interaction with one another [83]. Therefore, either other ET receptors or isoforms are activated in response to pathogen attack and/or ET pathway activation is related with other functions rather than pathogen defense only.

*ERF1* gene is a transcriptional factor of ET induced defense genes while it is an ET-responsive gene itself, being commonly used as an ET-pathway marker [79,84]. The *ERF1* gene was moderately induced in the resistant coffee variety contrasting with the increasing up-regulation in the susceptible variety, which culminates in a strong activation at the beginning of the necrotrophic phase. A comprehensive analysis of the ethylene role in plant response to pathogen attack was undertaken by Chen *et al*., (2003) [85]. When challenged with *C*. *destructivum*, the susceptible variety of *N*. *tabacum* produced ET in two distinct moments, the first during the biotrophic phase and the second during the necrotrophic phase (when leaf tissue was severely damaged or dead). It was then suggested that the first peak of ET production was related with the onset of unsuccessful defense responses and the second peak with the senescence of infected tissues. Although no ET production was measured in our assay, our results suggest that ET pathway activation in the susceptible variety may be related with tissue damage promoted by the fungal necrotrophic phase. In fact, it has been suggested that in later infection stages, hemibiotrohic pathogens may produce ethylene and deliver effectors or phytotoxins that manipulate the plant to produce ethylene for entering the necrotrophic stage of infection [86], and thus overcome plant defenses.

Overall, our results suggest that in both coffee varieties, the immune system enable the perception of the pathogen attack as the infection process starts, being the expression of resistance and susceptibility conditioned by the magnitude and/or timing of defense responses. In the resistant variety, the earlier and strong induction of JA biosynthesis and receptor-related genes, together with the activation of PR genes (*PR1* and *PR10*) seems to be important players in the set of defenses that resulted in the arrest of fungal growth. The stronger activation of the ethylene responsive-related gene *ERF1* at the switch to the necrotrophic fungal growth, that is coordinated with the activation profile of biosynthesis and receptor-related genes, suggests that this hormone may be relevant in susceptibility, although its possible involvement in defense responses is not discarded. To the best of our knowledge, this work represents the first attempt to unveil the involvement of phytohormones pathways in coffee-*C*. *kahawae* interaction making possible to engage on new exploratory functional assays. Future studies focusing on RNA-Seq analysis will provide a better understanding of the molecular mechanisms underlying the defense responses in this pathosystem.

## Supporting information

S1 Fig Schematic diagram of the current models of the SA, JA and ET pathways. a) SA pathway—SA is synthesized from chorismate through two distinct enzymatic pathways: PAL-mediated phenylalanine and ICS-mediated isochlorismate (IC). SA-induced redox changes lead to the reduction of inactive NPR1 oligomers to active monomers that are translocated into the nucleus, thus activating the defense-related genes (e.g. PR1); b) JA pathway–upon release from the chloroplast membrane, α-linolenic acid is converted into OPDA by sequential steps catalyzed by lipoxygenase (LOX), AOS and AOC. OPDA migrates into the peroxisome where, after reduction by OPR3 and three rounds of β-oxidation, (+)-7-iso-JA and its derivative (−)-JA is formed. By the action of JAR1 these last compounds are converted in the bioactive molecule JA-Ile. JA-dependent gene activation involves the JA-Ile binding to the receptor COI1. JAZ protein, which interacts with the SKP1-Cullin- F-box complex (SCF^COI1^) complex, is targeted for degradation by the 26S proteasome, releasing the transcriptional factor MYC2 and promoting the expression of JA-responsive genes (e.g. PR10); c) ET pathway–ET is synthesized from SAM in a two-step reaction catalyzed by ACS and ACO. In the absence of ET, the active CTR1 inactivates EIN2 and the phosphorylation of its C-terminal end is promoted resulting in suppression of the ethylene response. In the presence of ET, receptors like receptor ETR1 binds to the hormone becoming inactivated and, consequently, switching off CTR1. The C-terminal end of EIN2 is then cleaved off and migrates to the nucleus where it activates the expression of ethylene target genes, ERF1 included (adapted from [25,33]).
(TIF)

S2 Fig **Dissociation curves for non-specific qPCR products analysis of SA, JA, ET pathways related genes and qPCR reference genes:** a) *PAL*, b) *ICS2*, c) *NPR1*, d) *PR1*, e) *OPR3*, f) *COI*, g) *PR10*, h) *ACO2*, i) *ACS5*, j) *ETR1*, k) *EIN2*, l) *CTR1*, m) *ERF1*, n) *IDE*, o) *β-Tub9*, p) *S24*.
(PUB)

S3 Fig qPCR expression analysis of SA pathway associated genes. Relative expression pattern of a) *PAL/ICS2* (biosynthesis), b) *NPR1* (receptors), and c) *PR1* (responsive gene) obtained in Catimor 88 (R-resistant) and Caturra (S-susceptible) coffee varieties. Mean and standard deviation of three biological replicates is presented. Fold change as relative expression of gene expression between inoculated and control samples for each of the coffee varieties/inoculation time-points. Asterisks (*) represent statistical significance (p≤0.05) of gene expression between the two coffee varieties was determined by the non-parametric Mann–Whitney U test using IBM^®^SPSS^®^ Statistics version 20.0 (SPSS Inc., USA) software.
(TIF)
