# Supplementary figures and images for: A first insight into the involvement of phytohormones pathways in coffee resistance and susceptibility to Colletotrichum kahawae
Source: PLoS One. 2017 May 19;12(5):e0178159. doi: 10.1371/journal.pone.0178159 (PMC5438148; doi:10.1371/journal.pone.0178159)

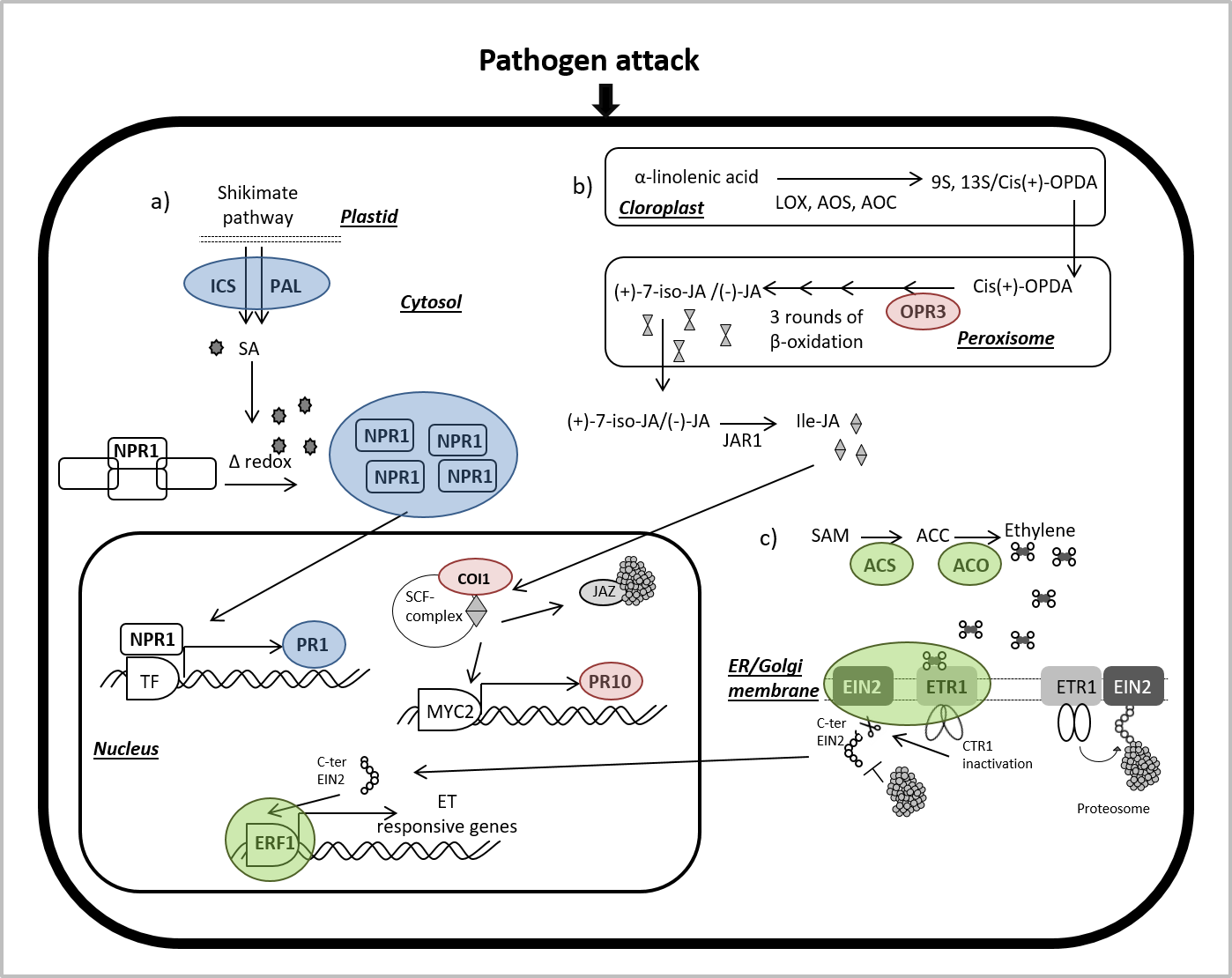

Supplement: S1 Fig — a) SA pathway—SA is synthesized from chorismate through two distinct enzymatic pathways: PAL-mediated phenylalanine and ICS-mediated isochlorismate (IC). SA-induced redox changes lead to the reduction of inactive NPR1 oligomers to active monomers that are translocated into the nucleus, thus activating the defense-related genes (e.g. PR1); b) JA pathway–upon release from the chloroplast membrane, α-linolenic acid is converted into OPDA by sequential steps catalyzed by lipoxygenase (LOX), AOS and AOC. OPDA migrates into the peroxisome where, after reduction by OPR3 and three rounds of β-oxidation, (+)-7-iso-JA and its derivative (−)-JA is formed. By the action of JAR1 these last compounds are converted in the bioactive molecule JA-Ile. JA-dependent gene activation involves the JA-Ile binding to the receptor COI1. JAZ protein, which interacts with the SKP1-Cullin- F-box complex (SCFCOI1) complex, is targeted for degradation by the 26S proteasome, releasing the transcriptional factor MYC2 and promoting the expression of JA-responsive genes (e.g. PR10); c) ET pathway–ET is synthesized from SAM in a two-step reaction catalyzed by ACS and ACO. In the absence of ET, the active CTR1 inactivates EIN2 and the phosphorylation of its C-terminal end is promoted resulting in suppression of the ethylene response. In the presence of ET, receptors like receptor ETR1 binds to the hormone becoming inactivated and, consequently, switching off CTR1. The C-terminal end of EIN2 is then cleaved off and migrates to the nucleus where it activates the expression of ethylene target genes, ERF1 included (adapted from [25,33]). (TIF) [file pone.0178159.s001.tif]

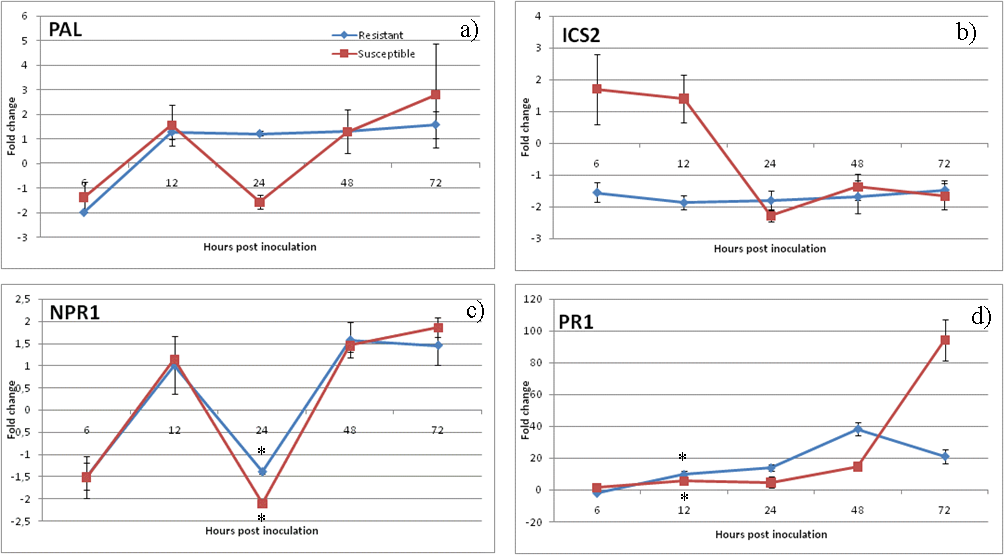

Supplement: S3 Fig — Relative expression pattern of a) PAL/ICS2 (biosynthesis), b) NPR1 (receptors), and c) PR1 (responsive gene) obtained in Catimor 88 (R-resistant) and Caturra (S-susceptible) coffee varieties. Mean and standard deviation of three biological replicates is presented. Fold change as relative expression of gene expression between inoculated and control samples for each of the coffee varieties/inoculation time-points. Asterisks (*) represent statistical significance (p≤0.05) of gene expression between the two coffee varieties was determined by the non-parametric Mann–Whitney U test using IBM®SPSS® Statistics version 20.0 (SPSS Inc., USA) software. (TIF) [file pone.0178159.s003.tif]
